# Adaptability as a Journey: A Constructivist Grounded Theory Study Exploring the Transition to Motherhood in the Context of Bipolar Disorder

**DOI:** 10.1177/10497323241297076

**Published:** 2024-12-05

**Authors:** Aigli Raouna, Andreea Miruna Mihut, Angus MacBeth

**Affiliations:** 1 Department of Clinical Psychology, School of Health in Social Science, 65932 The University of Edinburgh, Edinburgh, UK; 2 Edinburgh Law School, 34656 The University of Edinburgh, Edinburgh, UK

**Keywords:** perinatal bipolar disorder, pre-conception, pregnancy, motherhood, constructivist grounded theory

## Abstract

Despite growing evidence that women with bipolar disorder (BD) diagnoses are at a particularly increased risk for perinatal mental and physical health complications, our understanding of their experiences and support needs from pre-conception to early postnatal years is still in its early stages. To address this gap, a qualitative study was carried out employing a constructivist grounded theory approach to identify the underlying processes shaping women’s journeys to motherhood in the context of BD. In-depth, semi-structured online interviews were conducted with 10 mothers worldwide with a pre-existing diagnosis of BD and a first child under 5 years of age. Mothers’ experiences revolved around a constant interplay between vulnerability and adaptability, leading to the development of the substantive theory of adaptability as a journey. This study proposes that becoming adaptable constitutes a process, with the concept of “projecting adaptability” being influential in this journey. Characterized by the interconnected elements of self-awareness of vulnerability, perceived support from external sources, and ownership of experiences, the ability to envision an adaptable version of oneself along with understanding the path to achieving it played a significant role in women’s experiences. Overall, there is a need for a more dynamic understanding of these experiences, providing appropriate support rather than viewing women as simply vulnerable or adaptable. Further research is necessary to explore the transferability of this theoretical framework, especially among mothers from diverse socio-economic backgrounds.

## Introduction

Existing research consistently indicates that bipolar disorder (BD) poses unique challenges for women during the perinatal period, with potential implications not just for the women themselves but also for their children and their broader familial and social networks (Di Florio et al., 2018; Masters et al., 2022; Raouna et al., 2018; Sharma, 2022; Stapp et al., 2020; Stevens et al., 2019). Emerging evidence suggests that women with pre-existing BD are at heightened risk of obstetric complications (Solé et al., 2020), perinatal relapse, or exacerbation of mood episodes (Batt et al., 2022; Conejo-Galindo et al., 2022) and at higher risk of readmission to psychiatric units in the early postnatal period (Munk-Olsen et al., 2009). However, despite an increasing body of research advocating for better understanding and support for women with BD during this period (Batt et al., 2022; Demers et al., 2023), the literature demonstrates a noticeable imbalance. The current research landscape is largely dominated by a medical perspective, often overlooking the lived experiences and personal narratives of these women.

The decision to have children can present challenges for anyone, but, for individuals experiencing complex mental health difficulties such as BD, it often carries added layers of complexity (Dolman et al., 2013; Sahota & Sankar, 2020). Yet, research exploring these complexities is still remarkably sparse. A meta-synthesis of 23 qualitative studies on motherhood in the context of severe mental illness (including schizophrenia and BD) noted a primary focus on post-childbirth experiences, with scant attention on experiences during pregnancy or pre-conception decision-making processes (Dolman et al., 2013). The literature remains largely unchanged a decade after this review, with only a small number of studies exploring the experiences and support needs of women with severe mental illness during their transition to parenthood.

Among these few studies, Dolman et al. (2016) explored the factors influencing the decisions related to pregnancy and childbirth among women with BD at various stages of motherhood (i.e., considering pregnancy, currently pregnant, or previously pregnant). They interviewed 21 women and supplemented the data with information from an online forum in the United Kingdom. The key considerations in their decision-making process included the significance of motherhood, socio-economic factors, stigma, and fear. Similarly, a study in the Netherlands interviewed 15 women with BD who did not have children, revealing similar fears regarding the heritability of BD, the use of psychotropic medications during pregnancy, the risk of relapse, and doubts about their parenting abilities (Stevens et al., 2018). The narratives of women in this study also highlighted the invaluable nature of support from partners, family, friends, and professionals in navigating these challenges (Stevens et al., 2018).

Building upon these findings, Anke et al. (2019) conducted a study in Norway involving 17 women with BD who were either first-time or experienced mothers. They examined women’s concerns and preparations for parenthood, uncovering fears about the potential of relapse during pregnancy and postpartum and worries about the ability to take care of their child. Notably, this study also underscored the proactive measures taken by these women, such as organizing regular check-ups with healthcare providers and establishing supportive networks for the postnatal period, to balance their health worries with their aspirations of motherhood. Adding another dimension, Sahota and Sankar (2020) performed a text analysis of 1800 Reddit posts over the past 4 years from bipolar discussion boards, focusing on conversations around genetics and related topics such as reproductive decision-making. This study highlighted the complexity of these decisions, shaped by individuals’ past experiences, current circumstances, and future expectations, underscoring the value of shared experiences and peer support in navigating these challenges. Importantly, the central concept of “manageability” was also introduced, defined as individuals’ process of balancing their personal capacity and resources against the potential parenting challenges, including the risk of genetic transmission of BD to their potential children (Sahota & Sankar, 2020).

Parallel to the emergence of this valuable body of research, contemporary guidelines advocate for the management of BD via comprehensive, tailor-made plans that encompass the period from pre-conception through to pregnancy and the first postpartum year. These plans recommend the involvement of women and, when appropriate, their partners in complex decision-making processes related to breastfeeding and medication management, while being guided and closely monitored by interdisciplinary teams (Batt et al., 2022; Demers et al., 2023; Yatham et al., 2018). However, although these guidelines align with the support needs expressed by women with BD (Dolman et al., 2013, 2016; Stevens et al., 2018), it remains uncertain how effectively and consistently these guidelines are followed worldwide, given the primary context of existing studies being in Western, high-income countries. Additionally, the current research body has focused on specific aspects of the motherhood journey while neglecting others, leaving a gap in our understanding of the underlying processes that shape women’s experiences throughout their parenthood journeys.

To address these limitations, the purpose of this study was to develop a comprehensive theory that identifies the social processes influencing the transition to motherhood for women with pre-existing BD, focusing on the period from pre-conception through pregnancy to the early postnatal years. Specifically, this study sought to answer the question: What are the underlying processes and elements that shape women’s experiences as they transition to motherhood in the context of BD?

## Methodology

### Design

This study adopted a constructivist grounded theory approach (Charmaz, 2014) that assumes a relativist epistemology; acknowledges multiple standpoints, roles, and realities; adopts a reflexive stance; and situates the research in a social, situational, and historical context. This approach advocates that neither data nor theories are discovered. Instead, data and the subsequent theorizing are co-constructed by the researcher and research participants and filtered through the researcher’s background, values, assumptions, privileges, actions, and relationships with research participants (Charmaz, 2014). Compared to other well-established qualitative approaches, grounded theory was best suited for the current study as it facilitates the construction of a theoretical framework “grounded” in participants’ narratives, thereby making invisible processes transparent (Charmaz, 2017a). Furthermore, by employing a constructivist grounded theory approach, the researchers were less likely to impose their viewpoint on participants, amplifying participants’ voices and insights on experiences that usually remain hidden, unexpressed, or overlooked.

### Participants

#### Eligibility Criteria

Inclusion and exclusion criteria are presented in Table 1.

**Table 1.** Inclusion and Exclusion Criteria.

Inclusion Criteria	Exclusion Criteria
Over 18 years of age	Unable to give consent
Clinical diagnosis of bipolar disorder (any type) before becoming a mother (self-declared)	Not fluent in spoken English or the native language of the lead researcher
Having a first child up to 5 years old	Currently experiencing acute distress

#### Identification of Participants

A purposive sampling strategy was employed to recruit mothers from online community settings. Flyers and recruitment videos were promoted through non-profit organizations located worldwide that provide direct or indirect support to people affected by BD, online parenting groups, and mental health–related newsletters. All advertisements linked to a Qualtrics survey with detailed information about the study. Interested participants completed an eligibility screening form and, if eligible, were invited to consent to participating by agreeing to a set of statements and e-signing the form. All consenting participants were invited to provide an email address. Mothers also provided demographic information including age, current employment and marital status, higher education level attained, current region of residence, and child’s age.

In total, 44 individuals accessed the information sheet. Of those, 18 individuals signed the informed consent form and provided their contact details. Five potential participants did not respond to any email contact. Ten of the thirteen mothers who scheduled an interview attended, comprising the final study sample. Interviews were conducted by AR.

### Procedure

A semi-structured interview guide (Table S1) was used as a gentle guide for the areas to cover (Charmaz, 2015), informed by a review of related literature and developed in collaboration with academic and “experts by experience” consultees, including a young woman with a severe mental health diagnosis. A practice interview was conducted, enabling testing of content and pacing (Charmaz, 2014, 2015). The researcher adopted an intensive interviewing approach, whereby the interviews were co-constructed with participants, creating an open and interactive space in which the emphasis was placed on exploring each participant’s perspectives and experiences (Charmaz, 2014, 2015). Accordingly, the interview guide was used as a flexible tool, regularly revised throughout the research process by removing, rephrasing, or adding new questions. In line with the iterative approach of grounded theory and theoretical sampling principles, emergent concepts from previous rounds were tested and refined in the following rounds (Charmaz, 2014).

The first interview was conducted face-to-face in the home setting of the participant. All subsequent interviews were completed online via the University of Edinburgh Zoom domain. To ensure confidentiality, participants were asked to be in a quiet, private setting during the interview. Interviews were carried out in three rounds: November 2019 (*n =* 1), March–May 2021 (*n =* 6), and May–June 2022 (*n =* 3), averaging 75 minutes per interview (range: 49–111 minutes). All interviews were audio-recorded using an encrypted audio recorder. Each participant was offered a £10 (or equivalent) Amazon gift voucher and a study summary.

### Ethical Considerations

This project received ethical approval from the Department of Clinical and Health Psychology Ethics Research Panel at the University of Edinburgh (reference number: CLIN644). Due to the sensitive nature of the topics discussed and the online environment in which interviewing occurred, the lead researcher was especially attentive to any signs of distress that would warrant interruption or termination of the interview (Lobe et al., 2022).

### Reflexivity

A self-aware scrutiny of assumptions and alertness to how personal, social, and cultural background and beliefs may be influencing the research actions and relationships with participants was an integral part of the research process (Charmaz, 2017b; Dodgson, 2019). Within this study, the lead researcher assumed both the role of insider and outsider. Professionally, being an early career clinical psychology researcher with theoretical knowledge of developmental processes and ongoing research interests in the intergenerational pathways of BD contributed toward theoretical sensitivity (Charmaz, 2014). At a personal level, having lived experiences of BD family dynamics ignited the researcher’s interest on this topic and desire to amplify women’s voices whose support needs tend to stay unheard.

This emphasis on reflexiveness enabled the researcher to engage with participants and data analysis with an “open mind” (Charmaz, 2014). Among others, the cross-cultural nature of this project shed light on the fluctuation of power dynamics within the researcher/participant relationship, influenced by cultural and linguistic (a)symmetries (Au, 2019). Recognizing and understanding these subtle power imbalances during the interviews through memo writing highlighted the importance of contextualizing the experiences of mothers within broader social dynamics, especially considering that many of these women had navigated motherhood across multiple cultural contexts.

### Methodological Rigor

The lead researcher maintained a research journal to facilitate reflexivity and support the analytical process (Charmaz, 2014). Emotional responses and thoughts on sensitive topics discussed during interviews were reflected upon with the research team, supporting the lead researcher’s emotional availability to both negative and positive aspects of participants’ experiences (Melville & Hincks, 2016). To minimize biases and enhance credibility, the project employed triangulation. The third author independently coded three transcripts, and emergent themes and processes were thoroughly discussed in team meetings. Confirmatory bias was mitigated by examining and discussing any discrepant evidence. Extended participant quotes were included, allowing readers to form their own opinions on credibility and transferability (Hoyt & Bhati, 2007).

### Data Analysis

Following Charmaz’s steps (Charmaz, 2014), data analysis occurred concurrently with data collection, involving constant comparison of data, codes, concepts, and categories. After verbatim transcription of interviews, transcripts were coded and categorized, while the next round of interviews was scheduled. This cyclical process allowed emergent theoretical ideas to guide subsequent data collection. Memos were written alongside each step to track and advance abstract thinking. NVivo (version 12) was used to facilitate coding.

Transcription was followed by the initial coding stage, which involved breaking down the data into small units and inductively assigning them meaning while staying close to the data. Process coding (e.g., “taking control of the situation”) and in vivo coding (e.g., “it’s just go, go, go”) were used during this stage (Saldaña, 2021). Subsequently, focused coding involved grouping together initial codes with significant meaning and shared conceptual similarities or discharging irrelevant ones. Analytical memo writing was vital in supporting abstract thinking and “tolerating ambiguity” (Charmaz, 2006, p. 85) in this stage, comparing and conceptualizing emergent categories and their properties, dimensions, and nuances.

The fourth step included theoretical sampling and theoretical saturation. Acknowledging life’s complexity, which can never be fully captured in any theoretical framework (Apramian et al., 2017), and recognizing logistic constraints, recruitment ended once the research team judged that the inductive concepts were sufficiently developed and relationships between concepts were clear. The final step encompassed theoretical coding and diagramming relationships between categories to tell a coherent story (Charmaz, 2014). It progressed toward discovering the core category, “adaptability as a journey,” which encapsulated the underlying central process of transitioning to motherhood in the context of BD.

## Results

### Participant Characteristics

The characteristics of the 10 participating mothers are presented in Table 2.

**Table 2.** Participant Demographic Characteristics.

Mother	Age	Education Level	Employment Status	Marital Status	Current (and Past) Region of Residence	Gender of Child	Age of Child (in Years)	Bipolar Type
Mum 1	30–35	Graduate	Employed	Married	North Europe	Boy	1–3	I
Mum 2	30–35	Postgraduate	Unemployed	Married	Latin America	Boy	1–3	II
Mum 3	30–35	High school	Unemployed	In a relationship	North Europe	Boy	1–3	II
Mum 4	30–35	Graduate	Employed	Married	South Europe	Girl	1–3	II
Mum 5	25–30	Postgraduate	Employed	Married	North America (Central Asia)	Boy and Girl^a^	1–3 and <1	II
Mum 6	30–35	Postgraduate	Employed	Married	North America	Girl	1–3	II
Mum 7	35–40	Graduate	Unemployed	Married	South Europe	Girl	3–5	II
Mum 8	30–35	Postgraduate	Employed	Married	North Europe (South Europe)	Boy	<1	I
Mum 9	40–45	Postgraduate	Employed	Married	Oceania	Boy	3–5	II
Mum 10	35–40	Postgraduate	Employed	Married	North Europe (Latin America)	Girl	1–3	II

^a^Mum 5 had two children at the time of the interview.

### Adaptability as a Journey: The Role of Projecting Adaptability

The experiences of mothers transitioning to parenthood in the context of BD revolved around a constant interplay between vulnerability and adaptability, both ends representing a complex journey. All participating mothers faced adversities at various stages, from pre-conception to early parenthood, including reproductive, physical, mental health, relational, and parenting challenges—what are conceptualized here as vulnerabilities. However, the impact and outcome of these challenges varied both among women and within individual women along their motherhood journeys, constituting fluidity along the vulnerability–adaptability continuum rather than a linear trajectory toward one or the other. To understand how women negotiated these challenges and mobilized the resources at their disposal, the concept of “projecting adaptability” was developed, which played an influential role in shaping how mothers moved toward adaptability.

**Figure 1.**
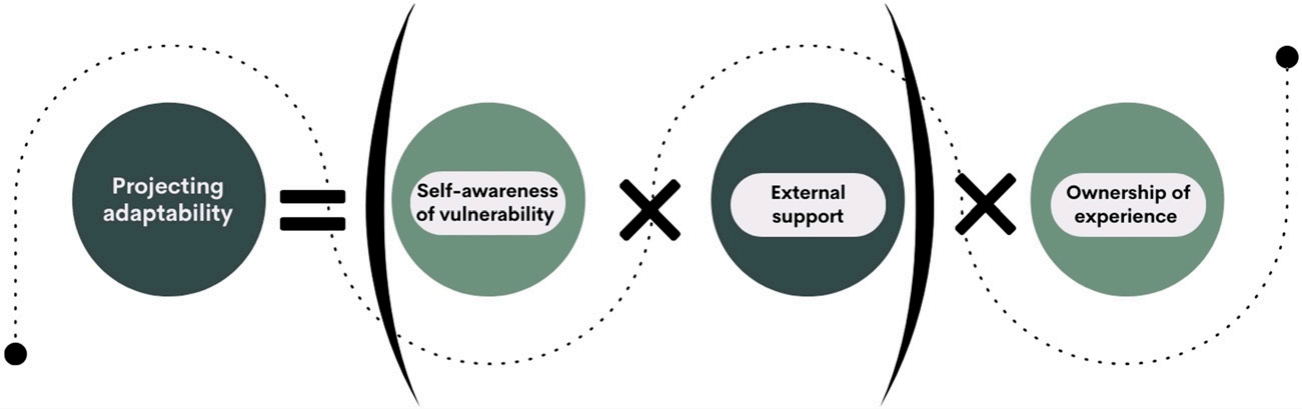
“Projecting adaptability” equation.

“Projecting adaptability” refers to the internal process of envisioning a potential version of oneself as capable to overcome adversity, understanding the path to achieving that capability, and working toward enacting that image. Although it is conceptualized as an internal process, it unfolds within the social context of each individual, characterized by the availability and accessibility of resources and support systems that can either enable or hinder adaptability. As such, projecting adaptability consists of three components: (1) self-awareness of vulnerability, (2) ownership of experience, and (3) perceived support from external sources (Table 3). These three elements, while visually represented as discrete entities (Figure 1), are not independent variables. As will become evident in the analysis, they are in constant interdependent interaction, with the presence of one functioning to compound the others—to different degrees across the motherhood journey.

**Table 3.** Summary of the Three Components of the “Projecting Adaptability” Concept.

Component	Summary
Self-awareness of vulnerability	Recognizing, understanding, and accepting one’s limitations and areas of weakness
Perceived support from external sources	Perceived availability, accessibility, and acceptability of support from informal (e.g., partner and peers) and formal (e.g., healthcare professionals) sources
Ownership of experience	Feeling involved in and in control of one’s life events, decisions, and circumstances, whether positive or negative; agency and proactiveness in changing/improving circumstances

The equation presented in Figure 1 serves to demonstrate the relationship between the component parts of the “projecting adaptability” concept rather than assigning numerical value to mothers’ experiences. In this equation, “self-awareness” is measured on a scale ranging between an absolute value of 0, representing a lack of understanding of the self’s vulnerabilities, limitations, and needs, and a positive number indicating a high understanding of these aspects. Perceived and received “external support” similarly ranges from 0, indicating the isolation of the mother’s experience, to a positive value. Meanwhile, “ownership” is projected as either existing (+1) or lacking (−1), giving the whole equation a valence toward adaptability (positive value) or vulnerability (negative value).

As the equation involves the multiplication of these key elements, if one of them is 0, then the entirety of the enterprise of projecting adaptability fails: it is not enough for the individual to be self-aware and take ownership of their experience if there is no external support; corollary, high levels of external support, and agency are misaligned without the direction given by self-awareness. Finally, as a concept, “ownership” differs from “self-awareness” in the sense that individuals may display a high level of self-awareness but without the agency and drive to enact change to perceived weaknesses; therefore, minus that agency, projecting adaptability is not possible. The dynamics of this concept are explored within the three stages of transitioning to motherhood: (1) Pre-conception; (2) Pregnancy and childbirth; and (3) Early postnatal period.

### Stage 1: Pre-conception

Before the first pregnancy [miscarriage] we talked about it a lot with my husband, I spoke to the psychiatrist about like breastfeeding on medication, taking medication at pregnancy, the pros and cons … I knew I wanted to have a baby so I wasn’t going to sway my decision, just how to go about it and the safest way for me. (Mother 1)

For most women, the passage from desiring to becoming a mother was far from linear. However, the desire to become a mother often provided a powerful sense of purpose and direction, guiding their decisions and actions amid various reproductive challenges and concerns intertwined with mental health–related issues. Their ability to project adaptability in the face of these challenges held an influential role in either moving women toward vulnerability or adaptability.

I’d always known I wanted to be a mom and I was kind of worried about passing it on to her, but I was like at least I’ll know what it is and be able to advocate for her to get meds sooner than I did and like with meds you can live a pretty normal life. (Mother 6)

Perceived accessibility and availability of resources, along with ongoing support from healthcare professionals, often served as the driving mechanism in assuming ownership and control of their experiences. In turn, this enabled them to recognize and embrace their personal and intergenerational vulnerability, and prepare themselves accordingly, both mentally and physically.

I knew I couldn’t like now, now [become pregnant], I needed to talk to my psychiatrist and they started, like, it took me for instance several months to leave the [medication], because I needed to decrease it and check that everything was okay. So I had regular checks during that period and it took me several months just to do that. (Mother 10)

However, when women’s desire to become a mother was not complemented by timely, consistent, and adequate professional and interpersonal support, their ability to project, and consequently enact, adaptability was compromised. Upon reflection, some women realized that their lack of pre-conception planning and support left them feeling incapable of influencing the negative cascading effects it had on other aspects of their lives, such as their relationship with their partners and overall well-being.

The hormone injections just made me really crazy and that really put a massive strain on our relationship, and I felt very distant from my partner and I wish we had been under the maternal mental health services … I really wish we had had help and plans in place before we became pregnant. (Mother 9)

Even when only one of the “projecting adaptability” equation elements was missing, women were put in a vulnerable position, demonstrating the interconnectedness of its components and the zero property of multiplication. Discrepancies in projecting adaptability, particularly in the self-awareness of vulnerability, reflected disparities in the accessibility of specialized knowledge, trust in healthcare systems, and the medical approaches of healthcare professionals, influenced by geographical and cultural factors. Through this lens, despite Mother 8’s access to external support and proactive intentions to plan for her motherhood journey, the absence of proper psychoeducation and (self-)awareness of vulnerability pushed her to the vulnerability end of the continuum.

I discussed with my psychiatrist that I would like to focus on my relationship, become a mother, so we decided to abruptly decrease [medication] which, I think, triggered my second [manic] episode, but also allowed me to truly understand my condition because I had been given the impression that I had a breakdown, that I would need to take this pill and everything else … I was not trained to see this condition as permanent, as something that can re-emerge … I didn’t have the necessary psychoeducation, neither did my partner nor those around me, so I remained exposed, and with the first reduction of [medication], I fell again. (Mother 8)

Regardless, vulnerability was not experienced as a static state. Despite the various challenges, some women took steps to regain control over their circumstances by negotiating and advocating for their needs. In certain cases, moving from vulnerability to projecting an adaptable self necessitated them to change to another care plan, care provider, or location.

Here [rural setting] it’s a general psychiatrist and he wasn’t actually that helpful, so I was really pleased I decided to go get my care at another area because the psychiatrist there was a lot more knowledgeable … just your luck really, a bit of a postcode lottery as to where the specialist services are. (Mother 1)

I was on [Medication 1] at the time, and the psychiatrist thought it'd be safer to try [Medication 2], so I tried [Medication 2] for a year and I was a mess, I was so anxious all the time it was pure torture, like I don’t know how I kept my shit together, but I was a disaster and so after a year I still wasn’t pregnant and so I said to my psychiatrist: “okay, this is not working, I can’t do this, like how much unsafe is [Medication 1] as opposed to the [Medication 2] I was on,” he said, “you know what, there’s a slightly higher risk for cleft palate, but it’s not as high of a risk, if you’re willing to do it, we can do that,” so I went back on [Medication 1] and I was so much happier. (Mother 6)

### Stage 2: Pregnancy and Childbirth

Mothers in this stage often struggled to project an image of themselves in the future that was both (i) adaptable while also (ii) a realistic version of themselves which factored in their particular vulnerabilities and lived experiences.

I felt devastated because I wasn’t living the “pregnancy dream.” (Mother 7)

The uncertainty led to mental constructions of absolute and reductive categories of “good mum” (i.e., adaptable, healthy, and ideal mum) and “bad mum” or “crazy mum” (i.e., vulnerable mum). In terms of external support, the relationship was ambivalent. Conversely, mothers demonstrated a yearning for this—in the form of professional expertise but also peers who went through a similar journey and adapted. On the other hand, the drive to avoid being a vulnerable mum often caused mothers to self-isolate from external support where this was perceived as untrustworthy.

There is a fair number of women here who’ve continued taking their medication for depression, but that doesn’t include things like atypical antipsychotics, mood stabilizers, stuff like that, so I would have liked to know someone who had done that, and I think, to really like know them personally … having a personal connection with someone … hearing that they had done it and it was fine, they’re fine, their kid is fine, that, would have been really reassuring for me. (Mother 5)

Now [that] I have this relationship with him [obstetrician], if I had another child I would be much more open but I didn’t know him and I was like “I’m not sure how much is …” you know, will they take the baby away from me if I tell them how bad I’m feeling? All that stuff. I was very resistant then, I wanted to be a good mum and I didn’t want to be a crazy mum. So I kind of tried to ignore that side of things. (Mother 9)

The struggle of self-awareness translated into a struggle to take ownership of their experience, characterized by uncertainty, paralysis, and wishful thinking. Especially when mothers felt criticized or sidelined by healthcare professionals in decisions about childbirth, breastfeeding, and practices in the neonatal intensive care unit (NICU), this resulted in a perceived loss of their parental “legitimacy.”

I had never considered the possibility of someone telling me not to breastfeed, I hadn't decided whether I wanted to do it or not, but it seemed like it was already decided for me. Of course, she [the gynaecologist] explained to me the reasons why it would be better not to breastfeed … and she was clearly right, but at the time I took it very hard, simply because I was not prepared for it ... I didn’t do it, but it took a toll on me as a mother. (Mother 7)

I had a really hard time agreeing to do Caesarean and I felt like, I just, I don’t know, silly really. At the time I felt judged, I thought I’m taking the easy way out if I have a caesarean, and the midwives in the hospital I felt were soo judgy and soo militant about breastfeeding and stuff, like there was no kind of leeway of different ways to become a mum, so I felt guilty saying yes. (Mother 9)

Commonly, the key to emerging from the state of vulnerable paralysis and wishfulness was dependent on the quality of external care. This is not to say that mothers were passive recipients of care; they often demonstrated agency and proactiveness in accessing this care and even changing their circumstances to access better care when this was lacking.

My mom had a traumatic birth experience … and I don’t know why, but people always scare you, so I was really scared, so I decided to actively try to change my mindset and go to this antenatal course with other parents. (Mother 10)

The nurses at the previous place [NICU] were just kind of rude and didn't make me feel confident in my ability to be a mom and a good mom [...] They dismissed a lot of my feelings because I'm mentally ill and crazy and whatnot in their opinion [...] at the second NICU they were just so much nicer and supportive and I got to have my space and time with her. (Mother 6)

Reductive, polar categories of “good mum” and “bad/crazy mum” were replaced by a much richer and more nuanced constellation of possible situations and categories. Mothers navigated these possibilities in creative ways, advocating for or creating contingency plans for each scenario. While a manifestation of the urge to take ownership of their experiences, this process was also self-perpetuating; as mothers grew in confidence and in their ability to project adaptability, the more they put plans in place for each eventuality collaboratively with trusted partners and healthcare providers.

My husband came along to the sessions with the psychologist and the psychiatrist and we had an in-depth discussion. We were somehow trained to be open to face all possible scenarios, somehow like having a decision tree … and my husband also knew what he should observe and what decisions should be made if I am not able to make decisions. (Mother 8)

Pregnancy is a hormonal onslaught like nothing else, so your previous strategies are really important, but you may need new ones […]. So I guess you can have a road map and two options or even a “okay, if I get to this point, consider this, if I get to this point,” you know, to have a well-informed, flexible plan in place that just takes the pressure off when you aren’t well or you’ve got a newborn baby that needs you 24/7. (Mother 9)

### Stage 3: Early Postnatal Years

Having a child constituted more than just adding a new variable into the equation for mothers; it represented an entire paradigm shift. Mothers tended to be highly self-aware of the monumental nature of the change and to acknowledge it as a watershed moment.

But now it’s always the child in the middle so ... you behave somewhat differently, you have this new parameter that forces you to try, you don’t always succeed, but try to keep it in your mind. (Mother 7)

For several participating mums, having the child brought new types and degrees of vulnerability. Tried-and-tested ways of coping with problems in the past were no longer an option, which in some cases had a severe negative impact on the well-being of mothers.

Normally if I was down, I wouldn’t see anyone, I wouldn’t go on my phone, I would just like stay in bed to sleep all day. But obviously now I’ve got a child so I can’t do that, at all, so I must force myself, and I think this is why I’m getting more and more depressed … I can show him I’m upset because I don’t want him to think the whole world is happy, but obviously I can’t be too upset so it’s like bottling up and then it’s draining me because I’m not getting that space I need. (Mother 3)

When external support was also lacking, the degree of vulnerability was compounded. Facing the failure of their usual coping mechanisms, mothers sought support in the form of external models in order to understand themselves. When this was not available, it led to impaired self-awareness, which often manifested itself as anxiety surrounding the reductive and absolute images of “good mum” and “bad mum” rather than the unique mum they were or could be.

General parenting strategies are helpful, but I would also like to know on a day when I can’t get out of bed like what do you do, how do you take care of your kid, like what are the strategies then, what is good enough parenting, like am I a failure of a parent if I give him a phone and he can just lay next to me and watch videos for an hour? (Mother 5)

I think we are very used to talk about the beauty of motherhood, but this part is really an ugly part and it’s really hard, rough to discover if it’s normal, or it’s a depression, or is just because we’re alone, and we’re supposed to be together being mothers. (Mother 2)

While navigating the complexities of nurturing an integrated yet evolving maternal identity, mothers highlighted the hidden motherhood realities and missed support opportunities. New mothers grappled with distinguishing between typical maternal distress and BD, with professional mental health expertise and support being more important than ever in this stage.

It wasn't until I was browsing online and I came across, I didn’t even know the term postpartum psychosis, like “Holy shit. I had that” and it explained my experience and my thought patterns exactly … And I was like, why did no one pick up on it? I was really angry. […] I’m angry at my doctor, and it’s affected my ongoing relationship with him because I don’t trust him as a doctor. (Mother 9)

However, as women transitioned into motherhood, another parallel, internal transformation also became apparent, related to their self-care intentionality. Women gradually became active agents who practice self-compassion and self-reflection. They acknowledged the importance of understanding and monitoring early signals and discussed their journey of feeling empowered to express feelings of unwellness and seek support before reaching a crisis point.

It’s hard for all mums, but I think it’s really hard for mums with bipolar because you’re worried about getting unwell and how will I look after a baby, and that can make you unwell cause you’re stressed .. just try to be honest with people about how you’re feeling, don’t bottle up, because that’s when problems happen with your mental health. (Mother 1)

Thus, in the early postnatal years, there was a massive upsurge in the willingness, ability, and practice of mothers taking ownership of their narrative. While having the child ostensibly constituted the new challenge to mothers’ journeys to adaptability, it also provided the powerful motivator for mums to overcome—not just new vulnerabilities but also older ones that they had not previously been able to take ownership over.

[Before our meeting] I didn’t know that I was more likely to get postpartum depression than other moms. And being aware of that I probably just tried to actively avoid getting depressed. You know, I’m feeling isolated, I shouldn’t be isolated, I just need to call someone and do something about it, because I don’t want to get postpartum depression. […] I’m actively avoiding it and because we made a plan, they made me write a plan of warning signs, what to do, and what to do about it and who should I talk to, and stuff like that. (Mother 10)

An increase in taking ownership prompted rises in the other elements of the equation—understanding themselves better, using external resources more. Far from conceptualizing their children as the cause of vulnerability, the child accelerated the process of “projecting adaptability” by prompting a heightened level of proactivity, fluidity, and even creativity, increasing all elements of the “projecting adaptability” equation.

How much of that is just being a sensitive mom, how much is, you know, a mood disorder is hard to say. But yes. In some ways more resilient, in some ways more volatile. It’s different, different. But I guess the biggest change is that I’m just so conscious of how my mood affects my family, my son … I can just see that immediate feedback, like “Oh, I’ve got to look after myself too, to be better for him.” (Mother 9)

Having navigated their own mood and emotional challenges, participating mothers were well-equipped to recognize and address similar issues in their children, demonstrating a heightened sensitivity to their children’s emotional states. Among others, pursuing professional help, openly discussing about feelings, and fostering emotional awareness through innovative strategies became part of their commitment to nurturing their children’s emotional health.

From a very young age we started talking a lot about emotions and she learned to name them. We also kept a feelings diary since she was about three years old … she’d narrate how her day went and I’d ask her “were you happy today, sad, did you get angry?” She’d choose a smiley face and tell me “today I got angry because so-and-so, today I laughed because” and that helped her to externalize her feelings, because it’s very important for me that she feels mentally and emotionally healthy. (Mother 7)

## Discussion

This study explored the underlying processes influencing the experiences of 10 women with pre-existing BD transitioning to motherhood, from pre-conception to the early postnatal years. Becoming vulnerable has been articulated as a process (Zarowsky et al., 2013)—a complex phenomenon rather than a singular concept when examined across the stages of pregnancy, birth, and the postnatal period (Briscoe et al., 2016). This study, viewed through mothers’ insightful narratives, proposes that becoming adaptable also constitutes a process, with the concept of “projecting adaptability” holding an influential position in this journey. Characterized by the interconnected elements of self-awareness of vulnerability, perceived support from external sources, and ownership of experiences, the ability to envision an adaptable version of oneself along with understanding the path to achieving it significantly shaped women’s transitioning to motherhood in the context of BD.

Viewing adaptability as a journey provides a heuristic framework in which vulnerability and adaptability are complex, fluid, and interactive qualities, with the ability of projecting adaptability being the catalyst of either moving individuals toward vulnerability or adaptability. This theoretical framework, grounded in women’s experiences, does not prioritize preventing unwanted situations (e.g., miscarriage and relapse) as the optimal outcome of transitioning to motherhood (i.e., eliminating vulnerability). Instead, the most valued transition outcome through this lens is the creation of a sustainable safety net to buffer against the adverse effects of these unwanted situations (i.e., projecting and enacting adaptability through awareness, agency, and trust in internal and external resources).

### Vulnerability as a Journey

The experiences of the mothers in the current study reflect and corroborate existing research, indicating the perinatal period as a time of heightened vulnerability for women with pre-existing BD (Di Florio et al., 2018; Masters et al., 2022; Sharma, 2022; Stevens et al., 2019). Mothers reported a wide array of challenges, from physical health complications and adjustments to severe mental health issues. Specifically, mental health complications covered the full continuum of mood episodes that characterize BD, from hypomania to mixed states, severe depression, mania, and postpartum psychosis, which in rare instances led to hospitalization and suicidal behaviors. While susceptibility to mood episodes during pregnancy and the postnatal period is well-documented (Conejo-Galindo et al., 2022; Di Florio et al., 2018; Masters et al., 2022; Sharma, 2022; Stevens et al., 2019), this study also shed light on largely unexplored experiences in this population.

Our findings emphasize the intertwined yet frequently compartmentalized facets of women’s well-being, encompassing mental, physical, and reproductive health. The experiences of mothers in this study resonate with existing evidence suggesting that women with BD face an increased risk for obstetric complications, such as labor induction, emergency or planned caesarean section, and neonatal complications like preterm birth or low birthweight (Runkle et al., 2023; Rusner et al., 2016; Solé et al., 2020). Yet, one alarmingly neglected area that featured prominently in mothers’ narratives was the association between reproductive outcomes and maternal mental health, especially in relation to fertility treatments, miscarriages, and monitored alterations in psychopharmacology. A recent scoping review, although not exclusively focused on BD, revealed a weak link between pre-existing maternal mental health conditions and infertility, which was found to contribute to experiences of additional distress during fertility treatments (Montagnoli et al., 2020). This review highlighted a critical need for preventive mental health strategies targeting women and couples undergoing fertility treatments, considering the known mental health risks associated with these procedures, particularly for those with complex mental health histories, which is further echoed by the findings of this study.

Additionally, mothers’ well-being was closely tied not only to their own health but also to the well-being of and emotional connection with their children, partners, and the broader environmental context. Half of the mothers in this study had babies requiring care in the neonatal unit, significantly impacting their mental health, particularly when met with dismissiveness and a lack of empathy from healthcare professionals. This finding aligns with Loewenstein et al.’s review (2019), underscoring the pivotal role of the NICU environment and communication (including the frequency, type, and tone of interactions with NICU providers) in directly influencing the psychological well-being of both parents. While a promising multilayered approach has been proposed for supporting parents and healthcare professionals in the NICU, incorporating trauma-informed (Sanders & Hall, 2018), family-centered (Treyvaud et al., 2019), and peer-support care (Fratantoni et al., 2022), the scalability and effectiveness of these strategies remain uncertain and further research is needed on understanding the NICU experiences and support needs of new parents.

Lastly, navigating motherhood amid present and past adversities introduced a unique set of challenges. Mothers described the challenges of managing their fluctuating moods and intergenerational traumatic experiences while striving to fulfil their parental duties and protecting their children from potential adverse exposure effects. The concept of “good enough” parenting, a recurrent concern illuminated in earlier qualitative studies (Anke et al., 2019; Dolman et al., 2013), became a pivotal question in this study as well, with mothers considering the potential distress caused to their children by mood inconsistencies and the dilemma of adequately explaining their mental health struggles to their young ones. This added dimension of complexity intensified mothers’ mental health issues in the early postnatal years. However, deterioration in mothers’ mental health in the perinatal period was often nested within wider stressors, including alterations in sleep patterns, feelings of isolation, and relationship tensions, confirming the multifactorial origin of mood episodes’ recurrence during this period (Batt et al., 2022; Demers et al., 2023; Sharma, 2022).

### Adaptability as a Journey

Amid vulnerability, mothers’ narratives in the current study also foregrounded the frequently overlooked power of adaptability. Vulnerability, a term often associated with potential harm or susceptibility to adverse events, has been conventionally viewed as a condition to be eliminated or minimized (Luthar et al., 2015). However, in the context of this study, vulnerability was not necessarily seen as a threat but rather a constant, a part of the human condition that needed to be acknowledged and navigated effectively. Through this lens, the challenges previously discussed, although significant, did not always represent an unmitigated crisis. Indeed, many of the mothers reported an increased sense of self-awareness, resilience, and adaptability in response to these challenges.

Earlier research has emphasized “manageability” as a key component in addressing prenatal and antenatal concerns, referring to the belief or perception that one has the necessary resources, support, and tools to cope with life’s challenges (Dolman et al., 2016; Sahota & Sankar, 2020; Stevens et al., 2018). In the realm of motherhood, manageability might involve a range of elements, from medication management and therapeutic support to social support networks and effective self-care routines (Demers et al., 2023; Sharma, 2022). Nevertheless, manageability is perceived as somewhat static and does not necessarily encapsulate life’s complex and dynamic nature. Consequently, this study proposes a progression of the concept from “manageability” to “adaptability.”

In this context, adaptability represents the capacity to adjust and respond flexibly amid the inherent uncertainties and challenges introduced by motherhood, specifically in the context of BD. Adaptability is a fluid concept that evolves over time, influenced by factors such as proactive engagement with change, learning from experiences, ongoing refinement of coping strategies, or strengthening support networks and healthcare support. Shifting from a problem-focused approach to a solution-oriented perspective enables women to actively shape their motherhood journey rather than managing the challenges along the way, aligning with recent clinical guidelines and policy recommendations (Demers et al., 2023; World Health Organization, 2022).

Furthermore, this study extends previous observational and experimental findings, introducing a more nuanced understanding. Existing studies have identified persistent difficulties in dyadic attunement, sensitivity, and responsiveness among mothers with BD, associated with sub-optimal socio-emotional development in their young children (Anke et al., 2019, 2020; Aran et al., 2021; Bjertrup et al., 2022). However, mothers’ narratives in this study uncovered a notable gap: the potential moderating role of awareness of these interactional patterns in guiding recovery actions. Mothers in this study displayed a heightened consciousness of their children’s potential vulnerabilities, to which they responded by implementing creative strategies, such as maintaining a “feelings diary” or proactively seeking professional help to foster their children’s emotional health. Whether mothers’ self-awareness and proactive strategies can effectively mitigate short- and longer-term socio-emotional risks for their children warrants further investigation.

### From Vulnerability to Adaptability: The Role of “Projecting Adaptability”

Experiencing and being conscious of “vulnerability” and “adaptability” does not imply that these are static categories; rather, with the introduction of agency and external support, the fluid category of “projecting adaptability” is created.

### The Role of Agency

Agency, or “taking ownership” of one’s narrative, was critical in women’s experiences during their transition to motherhood. Within this context, adaptive planning, a flexible approach facilitating strategic management of potential complications, emerged as crucial during this period. This corresponds with recent advocacy for pre-conception care and counselling for women with severe mental illness, including BD (Dolman et al., 2016; Sharma, 2022; Stevens et al., 2018). However, disparities in the consistency and universal application of guidelines, particularly in pre-conception planning and shared decision-making, were evident in this study, especially among participants beyond the Global North.

Many mothers expressed feeling excluded from decision-making, leading to defensiveness and resistance. Their narratives underscored the importance of not merely having preferences met but rather being given choices, access to information, and the opportunity to weigh pros and cons. The importance of agency was particularly evident in emergency situations, like emergency caesarean sections or NICU decisions. These findings align with research on perinatal experiences among mothers with complex mental health needs, such as opioid use disorder (Schiff et al., 2022) and postpartum psychosis (Roxburgh et al., 2023), emphasizing the centrality of agency and choice, as well as the need to feel in control, establishing boundaries aligned with their values and newly assumed maternal roles.

In this study, taking ownership of experiences in this productive way has been shown to not be an independent quality mothers have or do not have. Rather, the capacity to take ownership was enabled or disabled by a host of other factors, including degree of self-awareness and the presence of effective external support.

### The Role of Support

Consistent findings highlight the pivotal role of support, both formal and informal, during the transition to motherhood amid severe mental illness (Anke et al., 2019; Dolman et al., 2013; Mizock et al., 2019; Schiff et al., 2022). Echoing earlier research (Dolman et al., 2013; Schiff et al., 2022), mothers in the current study stressed the positive impact of supportive partners during challenging periods, also hinting at the unmet emotional and practical needs among partners. Although studies exploring the experiences of partners of women with BD during the transition to parenthood are lacking, qualitative research on the impact of postpartum psychosis on partners (Holford et al., 2018) and couples’ relationship (Wass et al., 2024) emphasizes existing gaps in supporting and equipping partners with the understanding and tools necessary for meaningful support while safeguarding their own mental well-being.

Another valuable source of support emerged from connecting with parents facing similar challenges. Peer-support mechanisms, well-received during pregnancy and postpartum, may offer a cost-effective solution to the current shortage of mental healthcare providers (Fang et al., 2022; Rice et al., 2022). Nonetheless, this study emphasizes the need for careful consideration to avoid predominantly favoring “well-functioning” service-user experiences, potentially overlooking more marginalized narratives and perpetuating “othering” feelings (Tambuyzer et al., 2014).

Equally, continuity of care and establishing trusting relationships with healthcare providers, especially interdisciplinary and specialist teams, were paramount to women’s mental health, resonating with earlier qualitative studies (Dolman et al., 2013; Schiff et al., 2022). Mothers stressed the significance of feeling comfortable enough to disclose distress symptoms without fear of judgment or dismissal, emphasizing the need for personalized and comprehensive care tailored to their unique needs and experiences. Insufficient, untimely care led to missed support opportunities during key motherhood stages, escalating symptoms to a crisis point and resulting in a “damage-control” support style that pushed women toward vulnerability.

### The Role of Self-Awareness

We also reiterate insights from earlier research on the multilayered prenatal and antenatal concerns shared by women with BD in balancing personal and societal expectations of “ideal motherhood” while facing or anticipating complications and risks related to BD (Anke et al., 2019; Dolman et al., 2013, 2016; Sahota & Sankar, 2020; Stevens et al., 2018). Nevertheless, the findings of this study also contribute a novel, optimistic perspective by unveiling an intentional transformation in mothers’ self-care behaviors and caregiving approaches along their motherhood journey.

This transition is underpinned by a gradual improvement in mothers’ reflective functioning capacity, often developed through their experiential understanding of the impact of their mood and actions on their children’s well-being. Heightened awareness of the interplay between mothers’ internal world and their children’s external behavior enabled better mentalization of their own thoughts, emotions, and needs, which in turn improved their comprehension of the motivations and intentions behind their children’s behaviors, fostering more attuned caregiving (Luyten et al., 2020). This finding holds promising implications for future interventions aiming at enhancing motherhood experiences in the context of BD. Further research is needed to explore how this approach can be leveraged to improve intergenerational well-being within complex perinatal mental health contexts (MacBeth et al., 2023).

### Limitations

There are several methodological considerations when interpreting the transferability of the proposed framework. The sample mostly comprises highly educated and well-resourced mothers with BD, not representing the full spectrum of experiences. Therefore, the findings provide only part of the overall picture, and the derived theoretical framework is context-dependent, subject to modification based on additional data (Charmaz, 2014). Additionally, although online interviewing allows researchers and participants to transcend geographical barriers, they can also pose challenges, including limited ability to fully capture non-verbal cues and a decreased sense of intimacy compared to traditional face-to-face interviews, potentially shaping interview content (Lobe et al., 2022). More research is needed to explore the transferability of the proposed theoretical framework, especially among mothers from diverse socio-economic backgrounds, using a multi-informant approach for a holistic understanding.

### Conclusion

The capacity to imagine a desirable self (in this case, an adaptable self) is a crucial first step in enacting the desired change (in this case, adaptability). The move from projecting to enacting has three crucial and interrelated components: that a mother has a level of self-awareness regarding her mental health, her weaknesses, her behaviors, and her coping mechanisms; that she perceives she has external support in the form of partners, family members, peers, and professionals and that this support is accessible to her; and that, ultimately, the combination of these elements enables her to take ownership of her narrative, to proactively make choices and changes to improve outcomes. It is through this equation that mothers can be empowered to move from simply being a “good mum” or a “crazy mum” to being their own kind of mum, complex and unique.

## Supplemental Material

Supplemental Material - Adaptability as a Journey: A Constructivist Grounded Theory Study Exploring the Transition to Motherhood in the Context of Bipolar Disorder Supplemental Material for Adaptability as a Journey: A Constructivist Grounded Theory Study Exploring the Transition to Motherhood in the Context of Bipolar Disorder by Aigli Raouna, Andreea Miruna Mihut, and Angus MacBeth in Qualitative Health Research

## References

[bibr1-10497323241297076] Anke T. M. S. Slinning K. Moe V. Brunborg C. Siqveland T. S. Skjelstad D. V. (2019). Mothers with and without bipolar disorder and their infants: Group differences in mother-infant interaction patterns at three months postpartum. BMC Psychiatry, 19(1), Article 292. 10.1186/s12888-019-2275-4 31533800 PMC6751750

[bibr2-10497323241297076] Anke T. M. S. Slinning K. Moe V. Brunborg C. Siqveland T. S. Skjelstad D. V. (2020). Bipolar offspring and mothers: Interactional challenges at infant age 3 and 12 months—A developmental pathway to enhanced risk? International Journal of Bipolar Disorders, 8(1), Article 27. 10.1186/s40345-020-00192-3 32869152 PMC7459000

[bibr3-10497323241297076] Anke T. M. Slinning K. Skjelstad D. V. (2019). “What if I get ill?” Perinatal concerns and preparations in primi- and multiparous women with bipolar disorder. International Journal of Bipolar Disorders, 7(1), Article 7. 10.1186/s40345-019-0143-2 30826916 PMC6397716

[bibr4-10497323241297076] Apramian T. Cristancho S. Watling C. Lingard L. (2017). (Re) grounding grounded theory: A close reading of theory in four schools. Qualitative Research, 17(4), 359–376. 10.1177/1468794116672914

[bibr5-10497323241297076] Aran P. Lewis A. J. Watson S. J. Nguyen T. Galbally M. (2021). Emotional availability in women with bipolar disorder and major depression: A longitudinal pregnancy cohort study. Australian & New Zealand Journal of Psychiatry, 55(11), 1079–1088. 10.1177/0004867421998796 33726546

[bibr6-10497323241297076] Au A. (2019). Thinking about cross-cultural differences in qualitative interviewing: Practices for more responsive and trusting encounters. The Qualitative Report, 24(1), 58–77. 10.46743/2160-3715/2019.3403

[bibr7-10497323241297076] Batt M. M. Olsavsky A. K. Dardar S. St John-Larkin C. Johnson R. L. Sammel M. D. (2022). Course of illness and treatment updates for bipolar disorder in the perinatal period. Current Psychiatry Reports, 24(2), 111–120. 10.1007/s11920-022-01323-6 35166993 PMC10339227

[bibr8-10497323241297076] Bjertrup A. J. Moszkowicz M. Egmose I. Kjærbye-Thygesen A. Nielsen R. E. Parsons C. E. Kessing L. V. Pagsberg A. K. Væver M. S. Miskowiak K. W. (2022). Processing of infant emotion in mothers with mood disorders and implications for infant development. Psychological Medicine, 52(16), 4018–4028. 10.1017/S0033291721000891 33866978

[bibr9-10497323241297076] Briscoe L. Lavender T. McGowan L. (2016). A concept analysis of women’s vulnerability during pregnancy, birth and the postnatal period. Journal of Advanced Nursing, 72(10), 2330–2345. 10.1111/jan.13017 27255232

[bibr10-10497323241297076] Charmaz K. (2014). Constructing grounded theory (2nd ed.). Sage.

[bibr11-10497323241297076] Charmaz K. (2015). Teaching theory construction with initial grounded theory tools: A reflection on lessons and learning. Qualitative Health Research, 25(12), 1610–1622. 10.1177/1049732315613982 26646825

[bibr12-10497323241297076] Charmaz K. (2017). Special invited paper: Continuities, contradictions, and critical inquiry in grounded theory. International Journal of Qualitative Methods, 16(1). 10.1177/1609406917719350

[bibr13-10497323241297076] Charmaz K. (2017). The power of constructivist grounded theory for critical inquiry. Qualitative Inquiry, 23(1), 34–45. 10.1177/1077800416657105

[bibr14-10497323241297076] Conejo-Galindo J. Sanz-Giancola A. Álvarez-Mon M. Á. Ortega M. Á. Gutiérrez-Rojas L. Lahera G. (2022). Postpartum relapse in patients with bipolar disorder. Journal of Clinical Medicine, 11(14), Article 3979. 10.3390/jcm11143979 35887743 PMC9319395

[bibr15-10497323241297076] Demers C. J. Walker R. Rossi N. M. Bradford H. M. (2023). Management of bipolar disorder during the perinatal period. Nursing for Women’s Health, 27(1), 42–52. 10.1016/j.nwh.2022.11.001 36528074

[bibr16-10497323241297076] Di Florio A. Gordon-Smith K. Forty L. Kosorok M. R. Fraser C. Perry A. Bethell A. Craddock N. Jones L. Jones I. (2018). Stratification of the risk of bipolar disorder recurrences in pregnancy and postpartum. The British Journal of Psychiatry, 213(3), 542–547. 10.1192/bjp.2018.92 30113284 PMC6429257

[bibr17-10497323241297076] Dodgson J. E. (2019). Reflexivity in qualitative research. Journal of Human Lactation, 35(2), 220–222. 10.1177/0890334419830990 30849272

[bibr18-10497323241297076] Dolman C. Jones I. Howard L. M. (2013). Pre-conception to parenting: A systematic review and meta-synthesis of the qualitative literature on motherhood for women with severe mental illness. Archives of Women’s Mental Health, 16(3), 173–196. 10.1007/s00737-013-0336-0 23525788

[bibr19-10497323241297076] Dolman C. Jones I. Howard L. M. (2016). Women with bipolar disorder and pregnancy: Factors influencing their decision-making. BJPsych Open, 2(5), 294–300. 10.1192/bjpo.bp.116.003079 27703792 PMC5013258

[bibr20-10497323241297076] Fang Q. Lin L. Chen Q. Yuan Y. Wang S. Zhang Y. Liu T. Cheng H. Tian L. (2022). Effect of peer support intervention on perinatal depression: A meta-analysis. General Hospital Psychiatry, 74, 78–87. 10.1016/j.genhosppsych.2021.12.001 34942447

[bibr21-10497323241297076] Fratantoni K. Soghier L. Kritikos K. Jacangelo J. Herrera N. Tuchman L. Glass P. Streisand R. Jacobs M. (2022). Giving parents support: A randomized trial of peer support for parents after NICU discharge. Journal of Perinatology, 42(6), 730–737. 10.1038/s41372-022-01341-5 35260824 PMC9184279

[bibr22-10497323241297076] Holford N. Channon S. Heron J. Jones I. (2018). The impact of postpartum psychosis on partners. BMC Pregnancy and Childbirth, 18(1), Article 414. 10.1186/s12884-018-2055-z 30352559 PMC6199718

[bibr23-10497323241297076] Hoyt W. T. Bhati K. S. (2007). Principles and practices: An empirical examination of qualitative research in the Journal of Counseling Psychology. Journal of Counseling Psychology, 54(2), 201–210. 10.1037/0022-0167.54.2.201

[bibr24-10497323241297076] Lobe B. Morgan D. L. Hoffman K. (2022). A systematic comparison of in-person and video-based online interviewing. International Journal of Qualitative Methods, 21(2). 10.1177/16094069221127068

[bibr25-10497323241297076] Loewenstein K. Barroso J. Phillips S. (2019). The experiences of parents in the neonatal intensive care unit: An integrative review of qualitative studies within the transactional model of stress and coping. The Journal of Perinatal & Neonatal Nursing, 33(4), 340–349. 10.1097/JPN.0000000000000436 31651628

[bibr26-10497323241297076] Luthar S. S. Crossman E. J. Small P. J. (2015). Resilience and adversity. In Lerner R. M. Lamb M. E. (Eds.), Handbook of child psychology and developmental science (7th ed., Vol. 3, pp. 247–286). Wiley.

[bibr27-10497323241297076] Luyten P. Campbell C. Allison E. Fonagy P. (2020). The mentalizing approach to psychopathology: State of the art and future directions. Annual Review of Clinical Psychology, 16(1), 297–325. 10.1146/annurev-clinpsy-071919-015355 32023093

[bibr28-10497323241297076] MacBeth A. Christie H. Golds L. Morales F. Raouna A. Sawrikar V. Gillespie-Smith K. (2023). Thinking about the next generation: The case for a mentalization-informed approach to perinatal and intergenerational mental health. Psychology and Psychotherapy: Theory, Research and Practice. Advance online publication. 10.1111/papt.12483 37534856

[bibr29-10497323241297076] Masters G. A. Hugunin J. Xu L. Ulbricht C. M. Moore Simas T. A. Ko J. Y. Byatt N. (2022). Prevalence of bipolar disorder in perinatal women: A systematic review and meta-analysis. The Journal of Clinical Psychiatry, 83(5), Article 21r14045. 10.4088/JCP.21r14045 PMC10849873 35830616

[bibr30-10497323241297076] Melville A. Hincks D. (2016). Conducting sensitive interviews: A review of reflections. Law and Method, 1(1), 1–26. 10.5553/REM/.000015

[bibr31-10497323241297076] Mizock L. Merg A. L. Boyle E. J. Kompaniez-Dunigan E. (2019). Motherhood reimagined: Experiences of women with SMI surrounding parenting. Psychiatric Rehabilitation Journal, 42(2), 105–112. 10.1037/prj0000339 30556726

[bibr32-10497323241297076] Montagnoli C. Zanconato G. Cinelli G. Tozzi A. E. Bovo C. Bortolus R. Ruggeri S. (2020). Maternal mental health and reproductive outcomes: A scoping review of the current literature. Archives of Gynecology and Obstetrics, 302(4), 801–819. 10.1007/s00404-020-05685-1 32671543

[bibr33-10497323241297076] Munk-Olsen T. Laursen T. M. Mendelson T. Pedersen C. B. Mors O. Mortensen P. B. (2009). Risks and predictors of readmission for a mental disorder during the postpartum period. Archives of General Psychiatry, 66(2), 189–195. 10.1001/archgenpsychiatry.2008.528 19188541

[bibr34-10497323241297076] Raouna A. Osam C. S. MacBeth A. (2018). Clinical staging model in offspring of parents with bipolar disorder: A systematic review. Bipolar Disorders, 20(4), 313–333. 10.1111/bdi.12604 29446217

[bibr35-10497323241297076] Rice C. Ingram E. O’Mahen H. (2022). A qualitative study of the impact of peer support on women’s mental health treatment experiences during the perinatal period. BMC Pregnancy and Childbirth, 22(1), Article 689. 10.1186/s12884-022-04959-7 36068490 PMC9450402

[bibr36-10497323241297076] Roxburgh E. Morant N. Dolman C. Johnson S. Taylor B. L. (2023). Experiences of mental health care among women treated for postpartum psychosis in England: A qualitative study. Community Mental Health Journal, 59(2), 243–252. 10.1007/s10597-022-01002-z 35900686 PMC9859833

[bibr37-10497323241297076] Runkle J. D. Risley K. Roy M. Sugg M. M. (2023). Association between perinatal mental health and pregnancy and neonatal complications: A retrospective birth cohort study. Women’s Health Issues, 33(3), 289–299. 10.1016/j.whi.2022.12.001 36621340 PMC10213085

[bibr38-10497323241297076] Rusner M. Berg M. Begley C. (2016). Bipolar disorder in pregnancy and childbirth: A systematic review of outcomes. BMC Pregnancy and Childbirth, 16(1), Article 331. 10.1186/s12884-016-1127-1 27793111 PMC5084442

[bibr39-10497323241297076] Sahota P. K. Sankar P. L. (2020). Bipolar disorder, genetic risk, and reproductive decision-making: A qualitative study of social media discussion boards. Qualitative Health Research, 30(2), 293–302. 10.1177/1049732319867670 31409193

[bibr40-10497323241297076] Saldaña J. (2021). The coding manual for qualitative researchers. Sage. https://digital.casalini.it/9781529755992

[bibr41-10497323241297076] Sanders M. R. Hall S. L. (2018). Trauma-informed care in the newborn intensive care unit: Promoting safety, security and connectedness. Journal of Perinatology, 38(1), 3–10. 10.1038/jp.2017.124 28817114 PMC5776216

[bibr42-10497323241297076] Schiff D. M. Work E. C. Foley B. Applewhite R. Diop H. Goullaud L. Gupta M. Hoeppner B. B. Peacock-Chambers E. Vilsaint C. L. Bernstein J. A. Bryant A. S. (2022). Perinatal opioid use disorder research, race, and racism: A scoping review. Pediatrics, 149(3), Article e2021052368. 10.1542/peds.2021-052368 35156121 PMC9044279

[bibr43-10497323241297076] Sharma V. (2022). Bipolar disorders. In Percudani M. Bramante A. Brenna V. Pariante C. (Eds.), Key topics in perinatal mental health (pp. 37–51). Springer. 10.1007/978-3-030-91832-3_3

[bibr44-10497323241297076] Solé E. Roca A. Torres A. Hernández A. S. Fernández N. Díaz C. N. Vieta E. Garcia-Esteve L. (2020). Obstetric complications in bipolar disorder: Psychiatric factors and the risk of caesarean section. European Neuropsychopharmacology, 32, 47–55. 10.1016/j.euroneuro.2019.12.115 31911063

[bibr45-10497323241297076] Stapp E. K. Mendelson T. Merikangas K. R. Wilcox H. C. (2020). Parental bipolar disorder, family environment, and offspring psychiatric disorders: A systematic review. Journal of Affective Disorders, 268, 69–81. 10.1016/j.jad.2020.03.005 32158009 PMC7175999

[bibr46-10497323241297076] Stevens A. W. Daggenvoorde T. H. van der Klis S. M. Kupka R. W. Goossens P. J. (2018). Thoughts and considerations of women with bipolar disorder about family planning and pregnancy: A qualitative study. Journal of the American Psychiatric Nurses Association, 24(2), 118–126. 10.1177/1078390317711251 28569088

[bibr47-10497323241297076] Stevens A. W. Goossens P. J. Knoppert-van der Klein E. A. Draisma S. Honig A. Kupka R. W. (2019). Risk of recurrence of mood disorders during pregnancy and the impact of medication: A systematic review. Journal of Affective Disorders, 249, 96–103. 10.1016/j.jad.2019.02.018 30769297

[bibr48-10497323241297076] Tambuyzer E. Pieters G. Van Audenhove C. (2014). Patient involvement in mental health care: One size does not fit all. Health Expectations, 17(1), 138–150. 10.1111/j.1369-7625.2011.00743.x 22070468 PMC5060706

[bibr49-10497323241297076] Treyvaud K. Spittle A. Anderson P. J. O’Brien K. (2019). A multilayered approach is needed in the NICU to support parents after the preterm birth of their infant. Early Human Development, 139, Article 104838. 10.1016/j.earlhumdev.2019.104838 31471000

[bibr50-10497323241297076] Wass N. Chadwick R. Caygill L. O’Mara O. (2024). “It kind of strips down your relationship to its defining features……it just kind of shone a light on what was already there”: A grounded theory of the impact of postpartum psychosis on the couple’s relationship. Journal of Reproductive and Infant Psychology, 42(2), 281–298. 10.1080/02646838.2022.2103793 35912867

[bibr51-10497323241297076] World Health Organization . (2022). Guide for integration of perinatal mental health in maternal and child health services. https://www.who.int/publications/i/item/9789240057142

[bibr52-10497323241297076] Yatham L. N. Kennedy S. H. Parikh S. V. Schaffer A. Bond D. J. Frey B. N. Sharma V. Goldstein B. I. Rej S. Beaulieu S. Alda M. MacQueen G. Milev R. V. Ravindran A. O’Donovan C. McIntosh D. Lam R. W. Vazquez G. Kapczinski F., Berk M. (2018). Canadian Network for Mood and Anxiety Treatments (CANMAT) and International Society for Bipolar Disorders (ISBD) 2018 guidelines for the management of patients with bipolar disorder. Bipolar Disorders, 20(2), 97–170. 10.1111/bdi.12609 29536616 PMC5947163

[bibr53-10497323241297076] Zarowsky C. Haddad S. Nguyen V. K. (2013). Beyond ‘vulnerable groups’: Contexts and dynamics of vulnerability. Global Health Promotion, 20(1 Suppl), 3–9. 10.1177/1757975912470062 23549696

