# Supplementary material for: Adaptability as a Journey: A Constructivist Grounded Theory Study Exploring the Transition to Motherhood in the Context of Bipolar Disorder
Source: Qual Health Res. 2024 Dec 5;35(12):1311–25. doi: 10.1177/10497323241297076 (PMC12379046; doi:10.1177/10497323241297076)
Supplement: Supplemental Material - Adaptability as a Journey: A Constructivist Grounded Theory Study Exploring the Transition to Motherhood in the Context of Bipolar Disorder [file sj-pdf-1-qhr-10.1177_10497323241297076.pdf]

Table S1: Interview guide example questions

Part of interview	Example Question
**Opening questions**	Would you like to start by telling me a little bit about you and your experience with bipolar disorder?
**Questions with a focus on before becoming a mother**	Focusing on the period before becoming parents, what did you know about having a baby as a woman experiencing bipolar disorder?
**Questions with a focus on pregnancy**	If you recall, could you describe how you found out that you would become a mum? What were your first thoughts and feelings when you found out?
**Questions with a focus on after childbirth**	Looking back at the first few months of your baby's life, are there any events that stand out in your mind? Could you describe them to me?
**Closing questions**	Based on your experience so far, what advice would you give to a woman with bipolar disorder and her partner who are thinking of starting their family?
